# A Case of Unilateral Facial Spasm With Vulnerable Hearing Function Due to a History of Cisplatin Treatment, Resulting in Intraoperative Hearing Loss

**DOI:** 10.7759/cureus.53695

**Published:** 2024-02-06

**Authors:** Akina Iwasaki, Masahito Kobayashi, Sachiko Hirata, Kazuhiko Takabatake, Masaki Ujihara, Takamitsu Fujimaki

**Affiliations:** 1 Neurosurgery, Saitama Medical University Hospital, Saitama, JPN

**Keywords:** auditory brainstem response (abr), ototoxicity, cisplatin, deafness, hearing loss, neurovascular compression, microvascular decompression, hemifacial spasm

## Abstract

A 51-year-old man with a history of cisplatin treatment for a right testicular tumor underwent microvascular decompression for hemifacial spasm. At an early stage in the surgical procedure, the intraoperative auditory brainstem response (ABR) was diminished despite a relatively minimally invasive approach, resulting in irreversible hearing loss. Cisplatin is known to cause dose-dependent hearing impairment primarily affecting the cochlea, but it can also induce neurotoxicity. In the present case, prior cisplatin administration may have caused fragility of the cochlear nerve as well. Patients with a history of ototoxic and neurotoxic drugs such as cisplatin require more careful manipulation and thorough intraoperative auditory monitoring during neurosurgical procedures that may affect hearing, such as those for hemifacial spasms.

## Introduction

Hemifacial spasm is a functional disorder characterized by intermittent and involuntary contraction of facial muscles due to vascular compression near the exit of the facial nerve. It can be cured by surgical release of the vascular compression (microvascular decompression [MVD]) [1]. A notable complication of this type of surgery is hearing impairment, which occurs in 2%-3% of cases [2]. The causes include stretching of the cochlear nerve due to retraction of the cerebellum, direct damage due to intraoperative manipulation or coagulation, and secondary ischemic changes due to anterior inferior cerebellar artery or small vessel injury. Recently, the incidence rate of complications associated with MVD appears to have improved with improvements in surgical techniques and intraoperative monitoring. A multicenter prospective study in Japan, which included data from our institution, found that only 0.6% of patients developed permanent hearing loss after MVD [3]. On the other hand, cisplatin and aminoglycoside antibiotics are well known to be ototoxic agents [4]. They cause dose-dependent hearing impairment, and several mechanisms have been reported including cochlear damage due to oxidative stress on hair cells, cochlear nerve neuropathy, or neurotoxic injury of the central auditory pathway [4-7].

 Here we document a patient with vulnerable hearing function possibly due to a history of cisplatin administration in whom a relatively less invasive procedure for MVD caused a rapid deterioration of the intraoperative auditory brainstem response (ABR) and hearing loss.

## Case presentation

A 51-year-old male patient presented to a primary care clinic with a complaint of left facial spasm. The symptom had become apparent in the lower eyelid in 2010, and by 2018, the spasm had spread to the left cheek and left corner of the mouth. Botulinum toxin treatment had been proposed by a former physician, but the patient preferred surgery and was referred to our hospital. At the age of 22, he had undergone high orchiectomy and cisplatin administration as postoperative chemotherapy for a right testicular tumor (embryonal carcinoma) at another hospital. Due to outdated records, the precise dose of cisplatin administered was unknown. When the patient visited our hospital, a left hemifacial spasm was evident, affecting the orbicularis oculi and orbicularis oris muscles. During facial spasms, the patient also experienced discomfort in the left ear, attributed to spasms of the stapedius muscle.

Findings of audiometry

The preoperative pure tone averages (average hearing thresholds at 500, 1000, 2000, and 3000 Hz) were 10.0 dB in the right ear and 13.8 dB in the left ear, indicating normal hearing function. However, hearing levels decreased to more than 40 dB at 4,000 Hz in both ears, and in the left ear, the hearing level at 6,000 and 8,000 Hz decreased to 25 and 30 dB, respectively. Word intelligibility was 100% bilaterally, and tympanometry demonstrated type A, indicating a normal middle ear. The preoperative ABR showed no obvious abnormalities. MRI showed a tortuous left vertebral artery and compression of the root exit zone of the facial nerve by the left anterior inferior cerebellar artery.

Intraoperative findings

**Figure 1:**
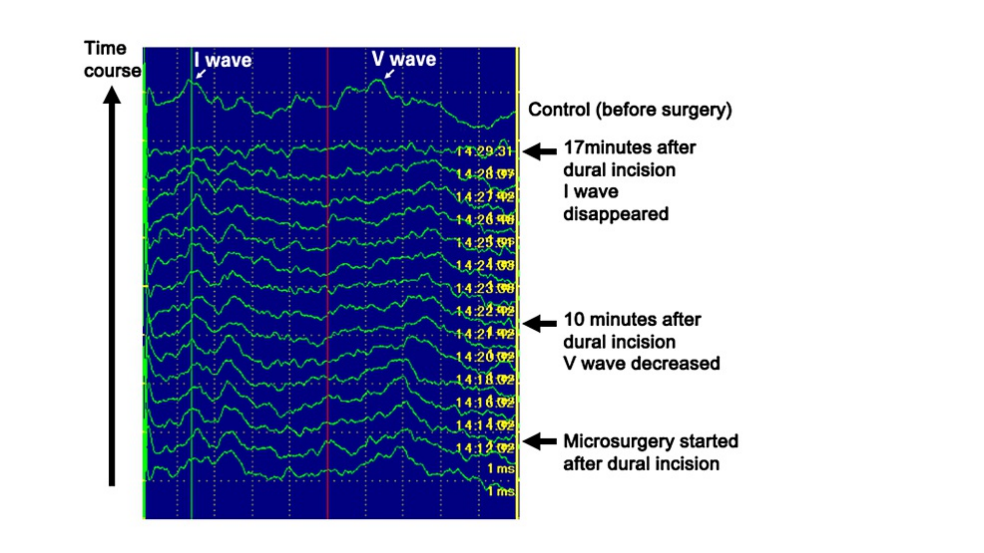
Intraoperative ABR. ABR, auditory brainstem response

**Figure 2:**
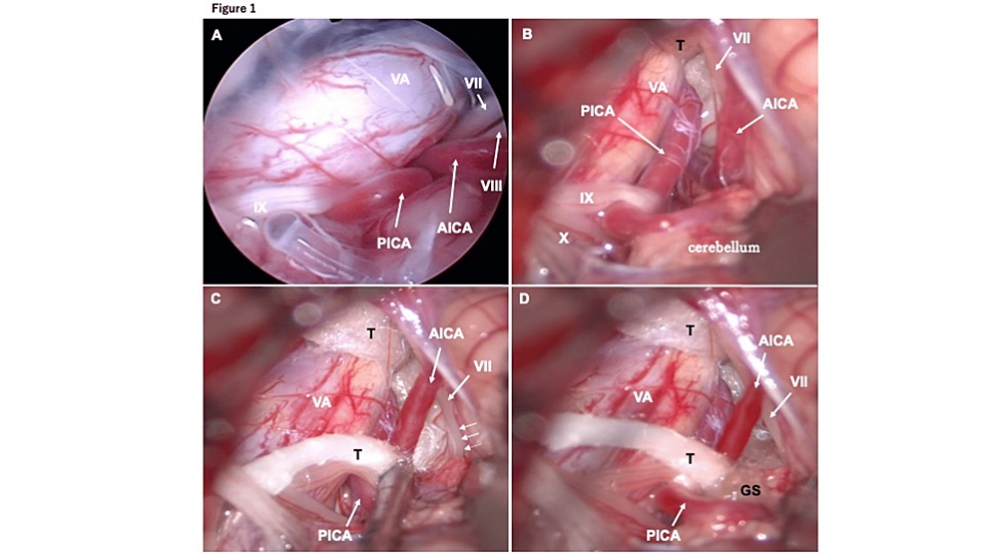
(A) Intraoperative endoscopic view; (B)-(D) microscopic views. (A) The left anterior inferior cerebellar artery (AICA), pushed by the vertebral artery (VA), compresses the exit zone of the facial nerve (VII). (B) The cerebellum was retracted, and the VA was moved ventrocaudally (left side of the image) using a hanging Teflon tape (T). The AICA is seen to compress the facial nerve exit zone. (C) Teflon tape (T) was draped over the AICA and moved caudoventrally (left side of the image). The facial nerve exit zone is indicated by the small arrow. (D) An absorbable gelatin sponge (GS) was inserted between the Teflon tape and the facial nerve exit zone. PICA, posterior inferior cerebellar artery; VIII, vestibulocochlear nerve; Ⅸ, glossopharyngeal nerve; Ⅹ, vagus nerve

The patient was positioned in right lateral recumbency with the head nearly horizontal under general anesthesia. MVD was performed via a suboccipital craniotomy. During surgery, ABR was continuously monitored to preserve hearing. During the first half of the procedure, which usually causes no problems, the amplitude of the ABR V wave was decreased with drainage of cerebrospinal fluid (CSF), dissection of the arachnoid membrane, and mild retraction of the cerebellar hemispheres (Figure 1). Therefore, surgical manipulation was immediately stopped, and the surgical field was refluxed and irrigated with artificial CSF until the ABR had recovered as previously reported [8]. The surgery was resumed after ABR recovery, and the left vertebral artery and anterior inferior cerebellar artery were identified at the site of the facial nerve (Figures 2A-2B). The arachnoid over the VIII nerve was not removed completely to protect the nerve, and the nerve was observed continuously within the operative field (Figures 2B-2D). However, when the anterior inferior cerebellar artery was gently teased away from the facial nerve using a blunt and edgeless instrument, the latency and amplitude of the ABR V wave again became prolonged and decreased. The operation was interrupted again to wait for ABR recovery, but this was not achieved even after a 20-minute pause and irrigation. Surgery was then resumed, and the anterior cerebellar artery was moved with polytetrafluoroethylene (PTFE) (Figures 2C-2D), completing the surgical procedures as scheduled. However, by the end of the procedure, the ABR waveform had not recovered.

Postoperative course

Postoperatively, the hemifacial spasm was resolved without any facial nerve palsy, but the left hearing function was lost. Postoperative audiometry revealed loss of left hearing, and right stereotropic nystagmus was also evident. Postoperative head MRI showed no abnormality such as edema or infarction. A corticosteroid was administered, and the nystagmus was resolved, but the hearing loss remained unimproved. The patient was discharged home on the 13th postoperative day, and at the last follow-up point 19 months after surgery, the preoperative pure tone average (average hearing thresholds at 500, 1,000, 2,000, and 4,000 Hz) was more than 110.0 dB in the left ear.

## Discussion

Hemifacial spasm is caused by the compression of blood vessels, often arteries, at the root exit zone of the facial nerve. Although some reports indicate that arteriosclerosis may be a cause of blood vessel compression, many patients do not show actual arteriosclerotic changes, and instead bending of arteries or anatomical features formed during the prenatal stage have been suggested [9]. Although botulinum toxin treatment can be used to paralyze the facial muscles for relief of symptoms, surgical decompression of the offending blood vessels is the only fully curative option [2]. On the other hand, a small but significant number of complications can develop after surgery even with established and improved surgical techniques as well as appropriate intraoperative functional monitoring. A recent multicenter prospective study in Japan found that 1.9% of patients experienced transient hearing disturbance and 0.6% developed permanent hearing loss [3]. Another study found that postoperative hearing impairment occurred in 2%-3% of patients after MVD for hemifacial spasm [2]. Postoperative hearing disturbance is often considered to be reversible in the long term when the symptoms are slight or mild [10], but recovery is difficult in cases of severe hearing impairment or loss [11,12]. Causes of postoperative hearing impairment include stretching of the cochlear nerve due to traction of the cerebellum, direct damage due to intraoperative manipulation or coagulation, and secondary ischemic changes due to anterior inferior cerebellar artery or small vessel damage [13,14]. Recent reports have suggested that dysfunction of hair cells due to drilling noise [15] and changes in perilymphatic pressure in the cochlea due to CSF depletion or overfilling [16] may be etiologic factors, but no previous reports have documented a history of ototoxic drug treatment as a cause of postoperative hearing loss, as was the case in the present patient.

Ototoxicity refers to damage to the structure and function of the cochlear vestibular system caused by exogenous substances such as drugs, chemicals, and ionizing radiation. Ototoxicity includes cochleotoxicity, vestibular toxicity, and neurotoxicity, and 200-600 ototoxic agents have been reported [17]. Typical agents include antimicrobials (aminoglycosides), platinum drugs (cisplatin), loop diuretics, and aspirin preparations (Table 1) [18]. The incidence of hearing impairment attributable to cisplatin and aminoglycoside antibiotics is cumulative and dose-dependent [4], and with cisplatin, progressive sensorineural hearing loss from high frequencies is observed on pure tone audiometry, with a marked tendency for the hearing loss to appear at doses exceeding 400 mg/m^2^ [19]. Cisplatin is used as chemotherapy for various tumors including testicular tumors, as in the present case. For testicular tumors, three to four courses of BEP (bleomycin 30 mg/body, etoposide 100 mg/m^2^, and cisplatin 20 mg/m^2^ every three weeks) have been recommended since the late 1980s as initial chemotherapy depending on the stage and degree of invasion [20]. The mechanism of cisplatin ototoxicity has been reported to be cochlear damage, including hair cell oxidative stress [4,5]. Cisplatin-induced neurotoxicity has also been reported [7], and high-dose cisplatin administration can prolong the latency of the ABR peak I and V waves [5,6]. These reports suggest that the cochlear nerve itself would have been damaged by previous cisplatin administration in the present case and would have been vulnerable, rendering it especially sensitive to only minor, minimally invasive manipulation.

**Table 1:** Agents with ototoxic risk. Modified from [18]. NSAIDs, nonsteroidal anti-inflammatory drugs

Antibiotics	Antimicrobial	Aminoglycosides (Streptomycin, Amikacin, Gentamicin, Tobramycin, Plazomicin) Tetracyclines (Minocycline, Tetracycline, Doxycycline) Polypeptides (Capreomycin, Polymyxin B, Bacitracin) Glycopeptides (Vancomycin, Teicoplanin) Macrolides (Azithromycin, Clarithromycin, Erythromycin) Quinolones (Ciprofloxacin, Moxifloxacin, Norfloxacin)
	Antifungal	Ciclopirox, Itraconazole, Ketoconazole
	Antiprotozoal	Metronidazole
	Antituberculosis	Isoniazid, Isoniazid, Cycloserine
	Antimalarial	Quinine, Chloroquine
	Antiparasitic	Paromomycin
Antiviral		Mefloquine, Ritonavir, Rimantadine, Cidofovir
Antineoplastic		Platinum-based (Cisplatin, Oxaliplatin, Carboplatin) Vinca alkaloid (Vinblastine, Vincristine, Vinorelbine) Polypeptides (Cyclosporine) Glycopeptides (Bleomycin) Mechlorethamine, Paclitaxel, Docetaxel
Diuretic		Loop diuretics (Furosemide, Torsemide, Bumetanide) Thiazides (Hydrochlorothiazide, Chlorthalidone) Acetazolamide
NSAIDs		Aspirin, Diclofenac, Ibuprofen etc.
Antihypertensive		Calcium-channel blockers (Amlodipine, Nicardipine, Nifedipine, Diltiazem) α-blockers (Carvedilol, Terazosin, Prazosin) ACE/ARB (Enalapril, Valsartan)

Although the incidence of cisplatin ototoxicity has been reported to increase with age, renal dysfunction, inflammation, genetic mutations, and concomitant medications, there have been no clear results from large-scale studies [4]. In the present case, a patient with slight hearing dysfunction and a history of cisplatin administration developed hearing loss as a result of a relatively minimally invasive, early intraoperative maneuver. Blood flow disturbance could have occurred in the internal auditory artery due to tension just after CSF drainage and gentle manipulation of the anterior inferior cerebellar artery. Although these procedures are routine and conducted with caution, the possibility of cisplatin-related injury could not be ruled out. Although the exact dose and duration of the previous cisplatin treatment were unknown, it had likely caused fragility of the cochlear and auditory pathways, thus contributing to hearing loss. It is noteworthy that patients with a history of exposure to ototoxic agents, even many years previously, may have acquired long-standing vulnerability in terms of hearing function. Patients with mild hearing dysfunction and a history of exposure to ototoxic or neuropathic drugs such as cisplatin require more careful surgical manipulation, thorough intraoperative nerve monitoring, and adequate informed consent regarding MVD for hemifacial spasm and other forms of neurosurgery and spinal surgery.

## Conclusions

We presented a case of a patient with a history of cisplatin administration, where a relatively minimally invasive surgical procedure around the auditory nerve led to significant hearing impairment. Considering that cisplatin can induce both neurotoxicity and cochleotoxicity, the cochlear nerve was likely susceptible due to the prior cisplatin exposure in this patient. Patients with a history of treatment involving ototoxic or neuropathic drugs, such as cisplatin, who undergo neurosurgery and spinal surgery, necessitate meticulous surgical manipulation and intraoperative nerve monitoring.
